# Bioengineering toward direct production of immobilized enzymes: A paradigm shift in biocatalyst design

**DOI:** 10.1080/21655979.2017.1325040

**Published:** 2017-05-19

**Authors:** Fabian B. H. Rehm, Shuxiong Chen, Bernd H. A. Rehm

**Affiliations:** a Institute for Molecular Bioscience, The University of Queensland, St Lucia, Brisbane, Australia; b Institute of Fundamental Sciences, Massey University, Palmerston North, New Zealand; c Australian Institute of Innovative Materials, University of Wollongong, Australia

**Keywords:** biocatalyst, biopolymer, enzyme, immobilization, polyhydroxyalkanoate, self-assembly

## Abstract

The need for cost-effectively produced and improved biocatalysts for industrial, pharmaceutical and environmental processes is steadily increasing. While enzyme properties themselves can be improved via protein engineering, immobilization by attachment to carrier materials remains a critical step for stabilization and process implementation. A new emerging immobilization approach, the *in situ* immobilization, enables simultaneous production of highly active enzymes and carrier materials using bioengineering/synthetic biology of microbial cells. *In situ* enzyme immobilization holds the promise of cost-effective production of highly functional immobilized biocatalysts for uses such as in bioremediation, drug synthesis, bioenergy and food processing.

## Introduction

Enzymes are diverse natural catalysts with the ability to perform reactions with high specificity and stereoselectivity, making them of great interest for a range of industrial processes as well as other applications, such as in bioremediation.^1^ However, their performance is significantly impacted by the surrounding environment, limiting the use of enzymes, which function optimally under the milder conditions of their native systems, in harsher and changing process environments. To overcome this, several bioengineering approaches have been undertaken. Rendering enzymes insoluble via immobilization is one such approach and generally aims to increase enzyme stability and reusability in continuous bioprocesses while retaining catalytic activity. While a range of immobilization strategies have been developed over recent years, the physical properties of the engineered biocatalysts need to be evaluated in the context of each process, and the most economically favorable option needs to be determined.^2-4^ These immobilization strategies can be broadly categorized into multi-step *in vitro* and one-step *in situ* approaches as well as carrier-based and carrier-free approaches.^5^

The *in vitro* approaches toward enzyme immobilization include enzymatic/chemical cross-linking or non-covalent adsorption to solid carrier materials. Alternatively, encapsulation, wherein the soluble enzyme is surrounded by a, often self-assembling, polymer carrier gel, could be used. The properties of the chosen carrier material are key determinants of the subsequent extent of improvement that the immobilization has provided, and thus the material most appropriate for the bioprocess conditions that is most compatible with the enzyme (while still remaining economically favorable) should be determined on a case-by-case basis. Notably, engineering the enzyme to increase compatibility with the carrier and process presents another option to achieve the desired biocatalyst characteristics.^6^ In contrast, carrier-free approaches leave less room for optimization and include enzyme-enzyme crosslinking (physicochemical or enzyme-catalyzed) and the addition of a translational fusion partner that interacts *in vitro*. Broadly, the steps involved in generating these immobilized biocatalysts can be categorized into first the production of the enzyme and then its immobilization.

**Figure 1.**
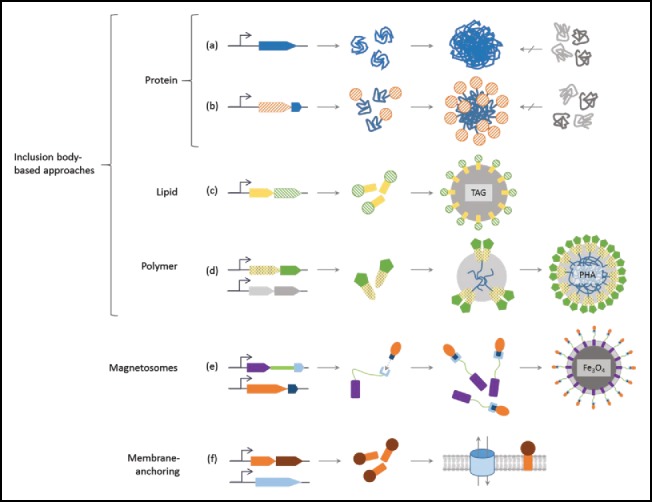
Strategies toward *in situ* enzyme immobilization. (a) Active protein inclusion body formation of the recombinantly overproduced enzyme (blue), non-homologous proteins (gray) are excluded from the aggregation. (b) An insolubility tag (blue) translationally fused to the enzyme of interest (orange, striped) results in pure protein inclusion bodies which display the enzyme. (c) Fusion of the target enzyme (green, striped) to PhaP1 (yellow), in the absence of polyhydroxyalkanoate (PHA) precursor synthesis by PhaA and PhaB, allows immobilization to triacylglycerol (TAG) inclusions. (d) Fusion of the PHA synthase PhaC (yellow, spotted) to the enzyme (green) while co-expressing the PHA precursor synthesis genes PhaA and PhaB (gray) allows for covalent immobilization to PHA inclusions. (e) Expressing a translational fusion of the magnetosome-anchoring protein Mms13 (purple) to the cohesin domain CohC (light blue) via a linker (green) while co-producing the enzyme to be immobilized (orange) translationally fused to the dockerin domain DocC (dark blue) allows immobilization to magnetosomes. (f) Producing a translational fusion of the enzyme to be immobilized (red-brown) to a membrane anchor (orange) allows for membrane-based immobilization (in this case, such that the enzyme is in the cytoplasm), co-producing lytic phage protein (light blue) allows for cytosol release and flow of reactants.

However, *in vitro* immobilization approaches are often met with economic concerns given their need for multiple production steps. In an effort to overcome such limitation, methods for one-step *in situ* immobilization present a lucrative alternative. Generally necessitating only the production and purification of the immobilized biocatalyst, these approaches avoid the often harsh, toxic, and/or expensive immobilization step. Additionally, the *in situ* immobilized state itself can sometimes facilitate ease of purification, further relieving economic concerns. Generally, existing cellular processes are used via a genetic approach. Means of *in situ* immobilization described thus far make use of protein, lipid, and polymer inclusion formation; magnetosomes; membrane vesicles; and insolubility tags (Fig. 1). As with the *in vitro* methods, the most suitable immobilization approach is likely process- and enzyme-dependent and should be assessed accordingly. Thus, as existing *in situ* immobilization approaches are further assessed and new developments are made, more options for optimizing *in situ* immobilized biocatalyst design will become available. Given the advantages that *in situ* methods present over their *in vitro* counterparts, we expect an increasing shift in focus toward their use in enzyme immobilization, a theme which will be expanded on in this commentary.

### Challenges associated with *in vitro* enzyme immobilization

Various immobilization methods have been established; however, each technique has its advantages and drawbacks. The most frequently used immobilization techniques are physical adsorption, entrapment, covalent attachment, and cross-linking.^7,8^

### Adsorption

Adsorption-based immobilization techniques attach enzymes to the carrier surface via weak forces, such as van der Waals forces, electrostatic forces, hydrophobic interactions, and hydrogen bonds.^9^ While enzymatic activity has been demonstrated to be retained in many cases, the immobilized enzyme prepared by adsorption can exhibit poor stability and enzymes can be easily stripped off from the carrier.^7,8^ Furthermore, effective biocatalyst preparation via this technique can prove challenging as the enzyme adsorption efficiency is highly susceptible to the immobilization parameters, including temperature, ionic strength, and pH.

### Entrapment

Immobilization via entrapment involves internalizing enzymes into polymer materials. For example, lipase from *Arthrobacter* sp. was immobilized by encapsulation in hydrophobic sol-gel materials. The encapsulated lipase showed increased stability and activity compared with the free form.^10^ A subsequent study also revealed that the encapsulated lipase had a higher activity than that of covalently immobilized lipase,^11^ presumably due to encapsulation preserving the mobility of the enzyme needed for enzyme activity.^8^ Nevertheless, encapsulation as non-covalent immobilization has weaker binding forces and hence potential release of the enzyme during repeated cycles of use.

### Chemical cross-linking

Covalent immobilization of enzymes uses cross-linking of non-essential pendant groups to the functional groups of the carrier material.^8^ The immobilization reaction to form the chemical bond is performed under mild conditions to retain enzyme function.^8^ However, in some circumstances, carrier materials do not provide functional groups, or the cross-linking reaction conditions are too harsh.^8,12^ Thus, to avoid compromising enzyme activity, immobilization carriers are often activated using functional reagents before immobilization, allowing for milder cross-linking conditions. For example, an approach to immobilize *Candida rugosa* lipase to chitosan used carbodiimide as coupling reagent to activate hydroxyl groups of the carrier.^12^ Generally, covalent binding-based immobilization provides a strong advantage by preventing enzyme shedding and leakage.^8^

To prevent steric hindrance in an effort to enhance enzyme activity, spacers/linkers may be inserted between enzyme and carrier.^8,13,14^ Enhanced enzyme activity is due to less structurally constrained display and improved accessibility of substrate.^8,13-15^

### The impact of carrier material properties on enzyme function

The structures and properties of the carrier materials strongly influence biocatalyst performance due to carrier-enzyme interactions and the generation of unique nano-environments wherein enzyme function needs to be assessed. Ideally, the chosen carrier materials should be nontoxic and biologically compatible.^16-18^ Natural polymer materials such as cellulose, chitin, chitosan and starch have been extensively studied as carrier materials as they are easy to modify, nontoxic, and generally compatible with enzyme function. They can be obtained from a wide range of sources, and have a variety of functional groups and good biocompatible properties.^17^ Furthermore, synthetic polymer materials, such as macroporous polyacrylamide microspheres, prepared by the chemical polymerization of various monomers have been demonstrated to be suitable carrier materials with strong mechanical rigidity and easily modifiable surface characteristics.^18^ In addition, magnetic particles have gained attention and act as carrier materials due to their intrinsic properties, including small particle size, excellent superparamagnetism, and large specific surface area.^19^

### Effect of enzyme loading

Excessive enzyme loading during the immobilization process often results in protein-protein interactions that can interfere with enzyme function by causing steric hindrance.^8,20^ For instance, one investigation on the immobilization of a pectinase showed that the activity declined when the loading amount of enzyme increased from 10 to 12 units/ml^18^. This decline in specific activity was also observed when overloading a carrier with lipase.^20^ Therefore, the amount of enzyme immobilized on carriers could affect the activity of the immobilized enzyme.^8^

### *In situ* immobilizations methods offer unique advantages

As process economics govern whether commercial realization of a given product can occur, it is paramount to determine the most cost-effective means of production. The use of enzymes in these processes is in itself an attempt at cost-reduction, and the use of immobilized forms of these enzymes to facilitate better process compatibility and reusability takes this a step further. The logical next step is to then pursue means of cost-reduction for the actual production of the immobilized biocatalyst. *In situ* enzyme immobilization strategies (Fig. 1) present a potentially major cost-reduction compared with the aforementioned *in vitro* approaches by eliminating the immobilization step; avoiding harsh/toxic conditions that could negatively impact enzyme activity by impacting structural integrity; and, in some cases, simultaneously easing purification steps.^5^ While entirely new *in situ* immobilization strategies or variations on existing ones are constantly being developed — a field of research with emerging interest — certain approaches will prove superior in terms of cost-efficiency and enzyme/process-compatibility.

### Protein inclusion bodies

The overproduction of recombinant proteins in bacterial hosts, such as *Escherichia coli*, can overload the relatively simple protein folding machinery.^21^ This results in aggregation of the folding intermediates into protein inclusion bodies throughout the bacterial cytoplasm. Interestingly, these inclusion bodies are pure in the recombinant protein^22^ and correctly folded forms may also be incorporated, leading to biologically active protein particles. Notably, an amorphous matrix fills the spaces between and inside the inclusion bodies, conceivably allowing diffusion of reactants. However, the characteristics of individual proteins greatly impact both whether the aggregates avoid degradation and whether active proteins (enzymes) can be incorporated. For proteins incompatible with this strategy, translational fusion to known active inclusion formers, such as PoxB,^23^ may be an indirect alternative for protein inclusion-based immobilization.

### Magnetosomes

To passively align along magnetic field lines, magnetotactic bacteria produce magnetosomes – membrane-enveloped magnetic nano-inclusions. By translationally fusing a target enzyme to a magnetosome-anchoring protein (e.g. MamC, MagA, Mms13, Mms16), immobilization can be achieved.^24^ The magnetic property of the magnetosomes subsequently allows for simple magnet-based isolation from the cell lysate, and later the reaction mixture. How compatible magnetosome-based biocatalysts are with a range of bioprocesses remains to be assessed.

### Cell membranes

As with the magnetosome immobilization approach, immobilization of enzymes to cell membranes has primarily been accomplished via translationally fusing the enzyme of interest to a membrane anchor. A more recent approach took this a step further i.e. following enzyme immobilization to the inner cytosolic membrane surface, lytic phage protein expression caused pore formation and release of the cytosol.^25^ The resultant cellular envelopes/membrane vesicles retained the enzyme and had overcome the severe mass transfer limitation of their whole cell biocatalyst counterpart, potentially making them suitable for process applications.

### Polymer/lipid inclusions

Under conditions of excess carbon availability, a range of bacteria produce insoluble storage inclusion bodies comprised of polymers such as the polyhydroxyalkanoates (PHAs) (e.g., poly(3-hydroxybutyrate, PHB) or lipids such as triacylglycerol (TAG).^26,27^ Generally, the hydrophobic inclusion core is surrounded by a protein shell and thus translational fusion of a protein to be immobilized to an inclusion-interacting protein has been the method of choice for *in situ* immobilization.^28^ In the case of TAG inclusions, there do not appear to be any highly abundant specifically associated proteins, but hydrophobic interaction-based immobilization has been achieved via fusion to PhaP1, a classically PHA granule-associated protein.^29^ In contrast, a variety of specific fusion partners are available for *in situ* immobilization onto PHA inclusions. While most interact hydrophobically, the PHA synthase, PhaC, such as the one from *Ralstonia eutropha*, remains covalently linked to the inclusion, providing a strong, highly oriented means of immobilization. By engineering the PHA biosynthesis pathway into industrial production hosts (e.g., *E. coli*), high yield one-step production of functionalized PHA granules for a variety of applications has been established.^30^ The use of PHA as the carrier provides a biocompatible, biodegradable, and versatile material platform making it compatible with a range of processes (including processes up to 100°C) and other applications such as bioremediation. Notably, PHA-immobilized enzymes have shown similar activities relative to their soluble counterparts while also possessing greater thermostability, longer storage stability, and greater reaction reusability.^31-35^ Furthermore, multi-enzyme immobilization for multi-step catalysis has been demonstrated.^36^

### Conclusions and future directives

In an era of growing demand for improved and stabilized biocatalysts, the *in situ* immobilization strategies offer an attractive alternative to the classical *in vitro* strategies. Inherently, by avoiding separate production of enzyme and carrier, production costs are proposed to be significantly reduced, enabling uses for high volume and low-cost conversion reactions. As attachment of enzyme to carrier occurs *in situ* in a permissive environment, a high level of functionality could be retained including the possibility of designing multi-enzyme arrays for cascade reaction as required for many processes such as for medical drug synthesis. Hence, *in situ* immobilization, such as the most extensively investigated PHA bead based approach, should be increasingly considered as a strategy for enzyme immobilization.
